# McGill University, Montreal

**Published:** 1893-12

**Authors:** 


MCGILL UNIVERSITY (MONTREAL).—FACULTY OF COMPARATIVE MEDICINE AND VETERINARY SCIENCE.

*Degree D. V. S. (Doctor of Veterinary Sciencc.)*

| | |
|---|---|
| Brainerd, E. - - - - | Kakoko, Mo. |
| Campbell, J. G. - - - | Montreal, P. Q. |
| Cleaves, A. S. - - - - | Rindge, N. H. |
| Dunton, H. B. - - - | Richmond, P. Q. |
| Denny, H. E. - - - - | New York City. |
| Lamb, A. S. - - - | Montreal, P. Q. |
| McGuire, W. C. - - - | Shawville, P. Q. |
| McDougall, J. - - - | Montreal, P. Q. |
| Orr, O. G. - - - | New Armagh, P. Q. |
| Plaskett, W. S. - - - | Woodstock, Ont. |
| Stephens, J. - - - | Huntingdon, P. Q. |
| Sturrock, T. - - - | Laggan, Ont. |
| Thayer, S. W. - - - | Cambridge, Mass. |
| Tracy, A. W. - - - | Sherbrooke, P. Q. |
| Wylie, M. C. - - - | New Harmony, Ind. |

